# Imaging of hepatic drug transporters with [^131^I]6-β-iodomethyl-19-norcholesterol

**DOI:** 10.1038/s41598-019-50049-8

**Published:** 2019-09-16

**Authors:** Masato Kobayashi, Kodai Nishi, Asuka Mizutani, Tsuzumi Hokama, Miki Matsue, Tetsuya Tsujikawa, Takeo Nakanishi, Ryuichi Nishii, Ikumi Tamai, Keiichi Kawai

**Affiliations:** 1 School of Health Sciences, Institute of Medical, Pharmaceutical and Health Sciences, Kanazawa University, Ishikawa, Japan; 2 Department of Radioisotope Medicine, Atomic Bomb Disease Institute, Nagasaki University, Nagasaki, Japan; 3 Biomedical Imaging Research Center, University of Fukui, Fukui, Japan; 4 School of Pharmaceutical Sciences, Institute of Medical, Pharmaceutical and Health Sciences, Kanazawa University, Ishikawa, Japan; 5 Faculty of Pharmacy, Takasaki University of Health and Welfare, Gunma, Japan; 6 Department of Molecular Imaging and Theranostics, National Institute of Radiological Sciences, Chiba, Japan

**Keywords:** Gall bladder, Preclinical research

## Abstract

We examined whether [^131^I]6-β-iodomethyl-19-norcholesterol (NP-59), a cholesterol analog, can be used to measure function of hepatic drug transporters. Hepatic uptake of NP-59 with and without rifampicin was evaluated using HEK293 cells expressing solute carrier transporters. The stability of NP-59 was evaluated using mouse blood, bile, and liver, and human liver S9. Adenosine triphosphate-binding cassette (ABC) transporters for bile excretion were examined using hepatic ABC transporter vesicles expressing multidrug resistance protein 1, multidrug resistance-associated protein (MRP)1-4, breast cancer resistance protein (BCRP), or bile salt export pump with and without MK-571 and Ko143. Single photon emission computed tomography (SPECT) was performed in normal mice injected with NP-59 in the presence or absence of Ko143. Uptake of NP-59 into HEK293 cells expressing organic anion transporting polypeptide (OATP)1B1 and OATP1B3 was significantly higher than that into mock cells and was inhibited by rifampicin. NP-59 was minimally metabolized in mouse blood, bile, and liver, and human liver S9 after 120 min of incubation. In vesicles, NP-59 was transported by MRP1 and BCRP. Excretion of NP-59 into bile via BCRP was observed in normal mice with and without Ko143 in the biological distribution and SPECT imaging. NP-59 can be used to visualize and measure the hepatic function of OATP1B1, OATP1B3, and BCRP.

## Introduction

Nuclear medicine imaging may potentially be used to directly detect and quantify the activity of drug transporters involved in drug clearance. In addition, imaging holds great promise in personalized medicine because it allows for characterization of individual patients before treatment initiation for a certain molecular target^1,2^. In clinical studies, [^99m^Tc]2-methoxyisobutylisonitrile and [^99m^Tc]tetrofosmin have been used for imaging of multiple drug resistance transporters in cancer before chemotherapy treatment^3^. For improved personalized medicine, accurate measurement of not only the drug resistance transporters but also the function of hepatic transporters is important because of inter-individual variability in plasma pharmacokinetics that controls drug clearance.

In the human liver, organic anion transporting polypeptide (OATP)1B1 (*SLCO1B1*), OATP1B3 (*SLCO1B3*), OATP2B1 (*SLCO2B1*), organic anion transporter (OAT)2 (*SLC22A7)*, organic cation transporter (OCT)1 (*SLC22A1*), and Na^+^-taurocholate cotransporting polypeptide (NTCP, *SLC10A1*) isoforms are mainly expressed at the sinusoidal membrane as solute carrier (SLC) transporters^4,5^. On the other hand, adenosine triphosphate (ATP)-binding cassette (ABC) transporters are mainly located on the bile canalicular or basolateral membrane in the liver. ABC transporters include multidrug resistance protein 1 (MDR1, P-glycoprotein, ABCB1 gene), multidrug resistance-associated protein 2 (MRP2, *ABCC2*), breast cancer resistance protein (BCRP, *ABCG2*), and bile salt export pump (BSEP, *ABCB11*) on the bile canalicular membrane and MRP1 (*ABCC1*), MRP3 (*ABCC3*), and MRP4 (*ABCC4*) on the basolateral hepatocyte membrane^4,5^.

During hepatobiliary scintigraphy with single photon emission computed tomography (SPECT), [^99m^Tc]diisopropyl iminodiacetic acid ([^99m^Tc]disofenin) undergoes high hepatic extraction via human OATPs^6,7^ and rapid bile canalicular excretion via human MRP1 and MRP2^8^. [^99m^Tc]3-bromo-2, 4, 6-trimethyl iminodiacetic acid ([^99m^Tc]mebrofenin) is taken up into the liver by human OATP1B1 and OATP1B3^9,10^, and is then excreted by MRP2^11^. Among the [^99m^Tc]pyridoxylaminates, [^99m^Tc]*N*-pyridoxyl-5-methyl-tryptophan is transported by human OATP1B1 and OATP1B3 and excreted by MDR1 and MRP2^12^. Clinical hepatobiliary SPECT scintigraphy rarely involves BCRP and/or BSEP. On the other hand, some positron emission tomography (PET) agents with ^11^C and ^18^F labeling are also delivered via drug transporters^13,14^. However, ^11^C-labeled PET agents cannot be used in general imaging centers because a cyclotron is required for synthesis of the PET agents.

Some glucocorticoid drugs and sulfate conjugates of estradiol and estrone, which are biosynthesized from cholesterol, are transported via BCRP^15^. In clinical nuclear medicine imaging, [^131^I]6-β-iodomethyl-19-norcholesterol ([^131^I]adsterol, [^131^I]NCL-6, NP-59), which is a cholesterol analog, is used to localize adrenal cortical lesions^16,17^. NP-59 is most useful for evaluation of incidentally detected adrenal adenomas and hyperaldosteronism. When considering the whole-body distribution, NP-59 prominently accumulates in the liver, and is then excreted into bile^18^. In this study, we examined if NP-59 was useful for measurement of the function of the hepatic BCRP transporter.

## Materials and Methods

### Materials

NP-59 was purchased from FUJIFILM Toyama Chemical Co., Ltd. (Tokyo, Japan). For evaluation of SLC transporters, rifampicin was purchased from Nacalai Tesque (Kyoto, Japan). For evaluation of ABC transporters, MK-571 sodium salt and Ko143 were purchased from Cayman Chemical (Ann Arbor, MI, USA) and Sigma-Aldrich (St. Louis, MO, USA), respectively. Human liver S9 was purchased from Corning Gentest (New York, NY, USA).

### Cells and vesicles

To investigate SLC transporters, human embryonic kidney (HEK)293 cells expressing human OATP1B1, OATP2B1, OATP1B3, OAT2, OCT1, or NTCP plasmid vector alone were prepared as described previously^19^. Briefly, HEK293 cells were transfected with the respective plasmid DNA, and were then selected with the appropriate antibiotics; cells were designated HEK293/OATP1B1, HEK293/OATP2B1, HEK293/OATP1B3, HEK293/OAT2, HEK293/OCT1, and HEK293/NTCP, respectively. We also tested mock-transfected cells. All cell lines were grown in Dulbecco’s modified Eagle medium (Wako Pure Chemical Industries Ltd., Osaka, Japan) supplemented with 10% (v/v) fetal bovine serum (Life Technologies, Carlsbad, CA, USA), 100 U/ml penicillin, and 100 μg/ml streptomycin at 37 °C in a 5% CO_2_ incubator.

To investigate ABC transporters, we used vesicles (GenoMembrane Inc., Kanagawa, Japan) without (control) and with high expression of human MDR1, MRP1, MRP2, MRP3, MRP4, BCRP, or BSEP. Experimental kits were also purchased from GenoMembrane Inc. and were used for experiments testing each ABC transporter.

### Partition coefficients

All our methods were performed in accordance with the relevant guidelines and regulations. Partition coefficients of NP-59 were measured using 2.0 ml n-octanol as the organic phase and 2.0 ml 0.1 M phosphate buffer (pH 7.4 for plasma) as the aqueous phase. n-Octanol and buffer were pre-mixed twice using a mechanical mixer for 1 min at room temperature. Then, 20 μl NP-59 in saline was added and mixed twice for 1 min at room temperature. Samples were centrifuged, and then the radioactivity of 200 μl of each phase was measured using a gamma counter. The partition coefficient was calculated by log(counts per min in n-octanol phase/counts per min in phosphate buffer). The partition coefficient of *N*-isopropyl-*p*-[^123^I]iodoamphetamine (^123^I-IMP) was used as a reference because ^123^I-IMP has high lipophilicity^20^.

### Transport assays with HEK293 cells and vesicles

Transport assays were performed according to our previously described method^12^. Before the transport assays with NP-59, we confirmed the transporter activity in HEK293 cells expressing each SLC transporter using [6,7-^3^H(N)]estrone-3-sulfate (PerkinElmer Inc., Waltham, MA, USA, 2.04 GBq/mmol, 2.22 TBq/mmol) for OATP1B1, OATP2B1, and OATP1B3; *p*-[^14^C]aminohippuric acid (PerkinElmer Inc., 2.04 GBq/mmol) for OAT2; [^3^H]methyl-4-phenylpyridinium (American Radiolabeled Chemicals Inc., St. Louis, MO, USA, 2.96 TBq/mmol) for OCT1; and [^3^H(G)]taurocholic acid (PerkinElmer Inc., 37 GBq/mmol) for NTCP. One day before the uptake experiments, HEK293 cells expressing a SLC transporter were plated at 4 × 10^5^ cells/well in 12-well plastic dishes. Cells were pre-incubated for 10 min in modified Hank’s balanced salt solution. Then, 37 kBq NP-59 with or without the OATP inhibitor, 1.0 mM rifampicin, was added to each well. At the end of the incubation, each well was rapidly washed twice with 1 ml ice-cold incubation medium. The cells were then solubilized in 0.5 ml 0.1 N NaOH, and radioactivity in an aliquot of the solution was measured with a gamma counter (AccuFLEXγ7000, Aloka, Tokyo, Japan). Subsequently, the protein concentration in the rest of the solution was measured using a BCA Protein Assay Kit (Thermo Fisher Scientific, Kanagawa, Japan). The accumulation rates of NP-59 in HEK293 cells were calculated as % injected dose (%ID) /g protein. Uptake of NP-59 into HEK293 cells expressing SLC transporters was compared with that in mock-transfected HEK293 cells. Assays were performed in octuplicate (n = 8 per HEK293 cells expressing each SLC transporter).

Excretion into bile was represented using ABC transporter vesicles transfected with MDR1, MRP2, BCRP, or BSEP. Excretion from liver into the blood was represented using ABC transporter vesicles transfected with MRP1, MRP3, or MRP4. Vesicles were pre-incubated for 10 min in reaction buffer. Each vesicle solution was incubated with NP-59 (37 kBq) for 5 min with adenosine triphosphate (ATP), which supplies energy for ABC transporters, or adenosine monophosphate (AMP), which does not provide energy and was used for comparison to ATP. At the end of the incubation, the vesicle solution was filtered through nitrocellulose filters, and the filters were rapidly washed twice with 1 ml ice-cold incubation medium. The vesicles were then solubilized in 0.5 ml 0.1 N NaOH, and the radioactivity was measured using a γ-ray counter (AccuFLEXγ7000, Aloka, Tokyo, Japan). Export of NP-59 from the vesicles into the ATP solution was compared with that in AMP solution. For the competitive inhibition assay, the vesicles were incubated with NP-59 for 5 min in the presence of inhibitor: final concentration of 50 µM MK-571 sodium salt for MRP and 50 µM Ko143 for BCRP^21–23^. At the end of the incubation, the vesicle solution was filtered through nitrocellulose filters, and the filters were rapidly washed twice with 1 ml ice-cold incubation medium. The vesicles were then solubilized in 0.5 ml 0.1 N NaOH, and radioactivity and protein concentration were measured as above. Assays were performed in octuplicate (n = 8 per vesicles expressing each ABC transporter). The accumulation rates of the vesicles of NP-59 were calculated as nmol/mg protein using the specific radioactivity of NP-59.

### Stability of NP-59 in mouse liver, mouse blood, and human liver S9

All animal studies were conducted following approval by the Animal Care Committee of Kanazawa University (AP-122339). Three ddy normal mice each (total of nine mice) were killed at 10 min, 30 min, and 60 min after injection of 370 kBq NP-59. After blood sampling via cardiocentesis, mouse livers and gall bladders were removed. Metabolites in mouse plasma, bile, and liver were analyzed with thin layer chromatography (TLC).

Human liver S9 was also used for metabolite analysis. Briefly, Krebs-Ringer phosphate buffer (pH 7.4) was added to the samples, followed by homogenization with an ultrasonic homogenizer (SONIFIER250, Branson, MO, USA). Then, ethanol was added to the homogenate to remove proteins, and the sample including blood was centrifuged for 5 min at 18,000 × *g*. The final supernatant was spotted onto the TLC plate, and the TLC spots were developed using hexane:ether:ethyl acetate at a ratio of 1:1:4^24^. After development and complete drying, the TLC plates were cut into 20 fractions, and the radioactivity associated with each fraction was measured using a γ-ray counter. The fractional ratios of metabolites were calculated by dividing the radioactive counts per minute (cpm) for each fraction by the total cpm.

### Biological distribution and SPECT imaging of NP-59 with and without Ko143 loading in normal mice

For the distribution experiments, normal ddy normal mice (male, 6 weeks old) were housed with a 12-hour light/12-hour dark cycle with free access to only water. Mice that had fasted all night were administered NP-59 via the tail vein (18.5 kBq/mouse). At 5, 10, 20, 30, 60, and 120 min, normal mice were euthanized under isoflurane (n = 5) anesthesia, and the following tissues were collected: blood, brain, thyroid, lung, heart, stomach, liver, gall bladder, small intestine, large intestine, kidney, adrenal gland, and bladder. Tissues were weighed, and radioactivity was quantified using a γ-ray counter to calculate the percent injected dose (%ID, radioactive cpm in tissue sample divided by cpm of injected radioactivity × 100) or percent injected dose per gram of tissue (%ID/g). Liver-to-blood, gall bladder-to-blood, and kidney-to-blood ratios were calculated by dividing %ID/g of liver, gall bladder, and kidney, respectively, by %ID/g of blood. For the inhibition study, mice were injected with about 1.0 mM (2.0 μg/g weight/100 μl) Ko143 immediately before injection of NP-59^22^. At 5, 10, and 30 min after injection, mice were euthanized under isoflurane (n = 5) anesthesia, and the same tissues were collected and quantified. Liver-to-blood, gall bladder-to-blood, and kidney-to-blood ratios were also calculated as above.

SPECT acquisition with a single pinhole collimator was started at 5 min after an intravenous bolus administration of NP-59 (about 5.0 MBq) using a micro-injection pump CFX1010 (ISIS, Osaka, Japan) for 15–20 sec into the tail vein of normal mice (n = 5) under 1.2–1.5% isoflurane anesthesia (Abbot Laboratories, Green Oaks, IL, USA) and continued for about 20 min with the step-and-shoot mode (20 sec/step) using a small animal SPECT/PET/computed tomography (CT) imaging scanner (TriFoil Imaging, Inc., Northridge, CA, USA). After SPECT imaging, CT scans (75 kV and 130 μA) were performed. The data were reconstructed using the maximum likelihood-expectation maximization with six iterations including attenuation and no scatter correction. The voxel size was set to 0.53 × 0.53 × 0.37 mm. Post-reconstruction smoothing filtering was applied using a 1.0-mm Gaussian filter. Image displays were obtained using medical image data analysis software called Amide’s a Medical Image Data Examiner, an open-source software tool (ver. 1.04). Transaxial and coronal images were displayed as maximum intensity projections. In these images, three to five regions of interest were placed over the liver and gall bladder with reference to CT images. Other normal mice (n = 5) were used for experiments using administration of about 1.0 mM Ko143, a BCRP inhibitor, immediately before injection of about 5.0 MBq NP-59.

### Statistical analysis

Data are presented as the means and standard deviation (SD). *P* values were calculated using the two-tailed unpaired Student’s *t* test for *in vitro* experiments after the F test, and the Wilcoxon rank sum test for *in vivo* experiments for comparison between two groups. A *P* value less than 0.05 was considered significant.

## Results

The partition coefficient of NP-59 was 1.62 ± 0.10, which was higher than that of ^123^I-IMP (0.94 ± 0.01) as a reference of lipophilicity.

**Figure 1:**
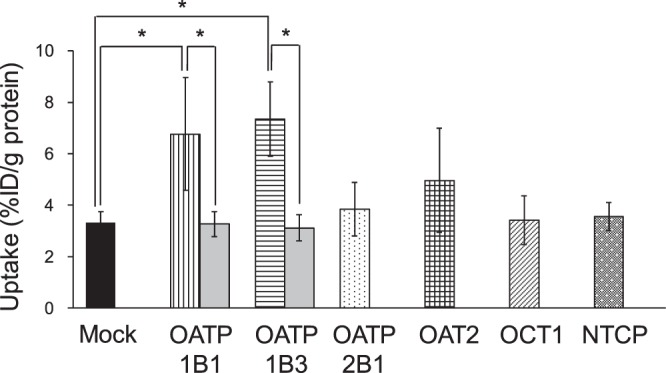
Uptake of NP-59 into HEK293 cells expressing SLC transporters. Uptake into HEK293/OATP1B1 and HEK/OATP1B3 cells was significantly higher than that in HEK293 mock cells. Uptake of NP-59 was also inhibited by rifampicin, an OATP inhibitor (gray bars). *P < 0.01 vs. HEK293 mock cells and the inhibitor (n = 8; unpaired Student’s *t* test).

Uptake of NP-59 into HEK293 cells expressing SLC transporters is shown in Fig. 1. Uptake of NP-59 into HEK293/OATP1B1 and HEK293/OATP1B3 cells was significantly higher than that into HEK293/mock cells (control) and that with rifampicin (*P* < 0.01, respectively).

**Figure 2:**
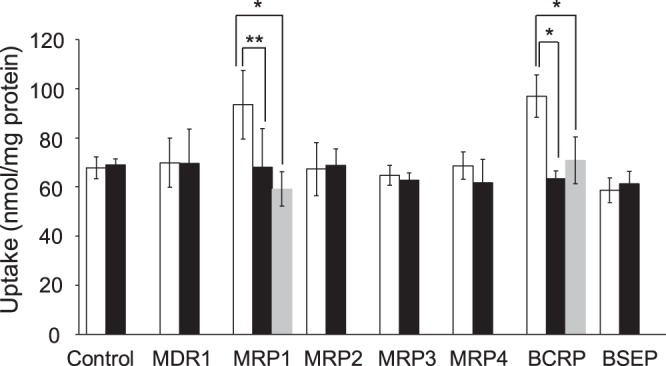
Uptake of NP-59 after 5 min of incubation in vesicles that highly express each ABC transporter. Uptake via MRP1 and BCRP in ATP solution (white bars) was significantly higher than uptake in AMP solution (black bars), and the uptake was blocked by MK-571 or Ko143 loading (gray bars). *P < 0.01 and **P < 0.05 between uptake in ATP and AMP solution and each inhibitor (n = 8; unpaired Student’s *t* test).

The stability of NP-59 in mouse blood, mouse liver, and human liver S9 is shown in Table 1. The rates of flow (Rf values) for NP-59 and [^125^I]NaI were in the ranges of 0.60–0.70 and 0.00–0.05, respectively, in hexane:ether:ethyl acetate. Although deiodination of NP-59 was identified, NP-59 had high stability in mouse plasma, bile, and liver, and human liver S9. Figure 2 shows the uptake of NP-59 in the presence of ATP or AMP solution into vesicles expressing hepatic ABC transporters (MDR1, MRP1–4, BCRP, or BSEP) after 5 min of incubation. We compared the uptake of NP-59 in ATP and AMP solution. Uptake into vesicles with high expression of MRP1 and BCRP in ATP solution was significantly higher than that in AMP solution and each inhibitor.

**Table 1:** Stability of NP-59 in mouse plasma, mouse bile, mouse liver, and human liver S9 fractions.

Time (min)	Mouse plasma	Mouse bile	Mouse liver	Human liver S9
10	95.4 ± 2.5	95.0 ± 3.8	95.1 ± 3.6	95.0 ± 4.3
30	94.8 ± 3.2	94.2 ± 4.2	93.5 ± 4.5	94.4 ± 2.1
60	93.9 ± 3.6	93.3 ± 3.5	92.8 ± 3.3	93.3 ± 3.2

The fractional ratios of NP-59 (non-metabolite) were calculated by dividing the radioactive counts for each fraction by the total radioactivity count.

Regarding the biological distribution of NP-59 in normal mice (Table 2), radioactivity in the blood, heart, liver, and kidney rapidly increased immediately after injection and then gradually decreased. Radioactivity in the lung and gall bladder also rapidly increased immediately after injection, continued to gradually increase by 60 min and 30 min, and then decreased. In the thyroid, stomach, small intestine, adrenal gland, and bladder, radioactivity gradually increased after injection. Small amounts of radioactivity were present in the brain and large intestine. In normal mice given NP-59 and Ko143 loading (Table 3), radioactivity in the blood, brain, liver, and kidney significantly increased compared to normal mice without Ko143 loading, whereas radioactivity in the gall bladder significantly decreased.

**Table 2:** Biological distribution of NP-59 in normal mice.

Organ (%ID/g) or %ID/organ)	5 min	10 min	20 min	30 min	60 min	120 min
Blood	141.89 ± 9.24	100.36 ± 9.15	88.65 ± 11.71	56.98 ± 9.09	54.30 ± 5.12	57.17 ± 6.97
Brain	0.27 ± 0.04	0.20 ± 0.04	0.18 ± 0.05	0.16 ± 0.00	0.16 ± 0.02	0.16 ± 0.03
Thyroid*	0.05 ± 0.05	0.10 ± 0.03	0.14 ± 0.02	0.16 ± 0.03	0.17 ± 0.10	0.20 ± 0.09
Lung	7.55 ± 2.17	10.21 ± 2.53	10.03 ± 0.68	10.67 ± 2.54	15.96 ± 4.89	12.93 ± 5.62
Heart	2.28 ± 0.42	1.98 ± 0.29	2.00 ± 0.33	1.97 ± 0.25	1.93 ± 0.31	1.69 ± 0.36
Stomach*	1.04 ± 0.23	0.80 ± 0.11	0.95 ± 0.17	1.05 ± 0.26	1.10 ± 0.21	1.13 ± 0.23
Liver	14.39 ± 0.89	15.93 ± 1.77	15.09 ± 1.87	13.75 ± 1.63	13.30 ± 1.35	11.53 ± 1.80
Gall bladder	12.98 ± 0.94	13.05 ± 1.08	23.20 ± 2.76	23.61 ± 2.87	14.80 ± 1.65	10.79 ± 1.25
Small intestine*	0.88 ± 0.07	0.99 ± 0.31	1.35 ± 0.24	1.74 ± 0.43	2.32 ± 0.52	3.11 ± 0.75
Large intestine*	0.27 ± 0.02	0.26 ± 0.08	0.26 ± 0.04	0.26 ± 0.05	0.25 ± 0.06	0.22 ± 0.08
Kidney	2.07 ± 0.37	1.88 ± 0.31	1.91 ± 0.28	1.88 ± 0.28	2.15 ± 0.05	2.26 ± 0.38
Adrenal gland	0.51 ± 0.12	0.80 ± 0.20	1.02 ± 0.28	1.15 ± 0.47	2.09 ± 0.32	3.05 ± 1.02
Bladder	0.53 ± 0.45	0.67 ± 0.61	1.33 ± 0.54	1.43 ± 0.91	1.50 ± 0.80	2.11 ± 0.88

%ID/g indicates percent injected dose per gram of tissue.

*%ID/organ was calculated from %ID per organ.

Values are the mean ± standard deviation obtained from five mice.

**Table 3:** Biological distribution of NP-59 with Ko143 loading.

Organ (%ID/g or %ID/organ)	5 min	10 min	30 min	60 min
Blood	165.51^†^ ± 18.31	124.42^†^ ± 10.82	70.36^†^ ± 10.68	60.96^†^ ± 10.06
Brain	0.34^††^ ± 0.00	0.28^††^ ± 0.02	0.24^††^ ± 0.01	0.20 ± 0.02
Thyroid*	0.06 ± 0.03	0.08 ± 0.03	0.11 ± 0.02	0.16 ± 0.08
Lung	6.73 ± 1.11	16.47 ± 8.05	12.25 ± 7.39	11.89 ± 5.12
Heart	3.13^†^ ± 1.12	2.76 ± 0.60	1.96 ± 0.21	1.92 ± 0.28
Stomach*	0.72 ± 0.31	1.08^†^ ± 0.22	1.56^†^ ± 0.28	1.21 ± 0.32
Liver	16.44^†^ ± 1.99	20.52^†^ ± 2.35	18.87^††^ ± 0.57	14.21 ± 2.12
Gall bladder	10.25^††^ ± 0.85	10.88^††^ ± 1.04	18.65^††^ ± 2.08	14.91 ± 1.27
Small intestine*	1.05 ± 0.23	1.15 ± 0.76	1.42 ± 0.64	2.28 ± 0.55
Large intestine*	0.37 ± 0.15	0.33 ± 0.12	0.36 ± 0.13	0.27 ± 0.12
Kidney	2.71^†^ ± 0.59	2.59^†^ ± 0.31	2.61^†^ ± 0.24	2.59^†^ ± 0.15
Adrenal gland	0.53 ± 0.10	0.84 ± 0.17	1.19 ± 0.21	2.55^†^ ± 0.23
Bladder	0.62^†^ ± 0.29	0.62 ± 0.56	1.57 ± 0.97	1.71 ± 1.02

%ID/g indicates percent injected dose per gram of tissue.

^*^%ID/organ was calculated from %ID per organ.

Values are the mean ± standard deviation obtained from five mice.

^†^*P* < 0.05 and ^††^*P* < 0.01 compared with normal mice.

**Figure 3:**
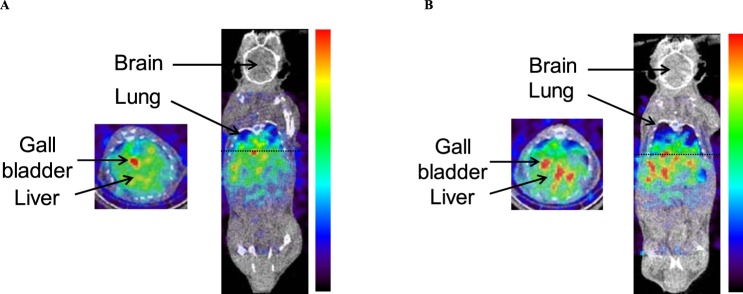
Whole-body SPECT images of normal mice without (**A**) and with Ko143 loading (**B**) under 2.0% isoflurane anesthesia injected with 5.0 MBq NP-59 at 5–25 min. The arrows show the gall bladder, and dashed lines show the level of the transaxial images. NP-59 accumulated in the lung, liver, and gall bladder in normal mice. In mice with Ko143 loading, accumulation of NP-59 in the liver was increased, whereas accumulation in the gall bladder was slightly decreased compared with normal mice.

Figure 3 shows SPECT images of a mouse at 5–25 min after injection of NP-59 without (Fig. 3A) and with (Fig. 3B) Ko143 loading. NP-59 primarily accumulated in the lung, heart, liver, and gall bladder in normal mice. In mice with Ko143 loading, accumulation of NP-59 in the liver increased from 14.0 ± 4.5 (normal mice) to 16.7 ± 3.8 counts (mice with Ko143 loading), and accumulation in the gall bladder slightly decreased from 33.9 ± 8.4 (normal mice) to 26.5 ± 10.9 counts (mice with Ko143 loading).

## Discussion

Measurement of hepatic transporter function is important for prediction of the effect of chemotherapeutic agents. In this study, we aimed to develop imaging of hepatic function using NP-59, a cholesterol analog. NP-59 was confirmed to be a lipophilic agent because it showed a higher partition coefficient than ^123^I-IMP, which is generally known as a lipophilic agent in nuclear medicine^20^. Due to its high lipophilicity, the radioactivity of NP-59 in the blood was higher than that in other tissues (Table 2) because about 74% of NP-59 binds to serum proteins in blood as shown with ultrafiltration in our previous study (unpublished observations). In addition, some NP-59 was non-specifically bound and/or adsorbed to the HEK293 cell surface membrane, vesicles, and the experimental apparatus (Figs 1 and 2) although the cells and vesicles were washed twice with 1 ml ice-cold incubation medium.

Regarding SLC transporters for liver uptake of NP-59, we observed involvement of OATP1B1 and OATP1B3 (Fig. 1). Although other SPECT and PET imaging agents used for hepatobiliary scintigraphy are also taken up via OATP1B1 and OATP1B3^6,7,9,10,12–14^, the liver uptake of NP-59 was slower than that of other agents because we estimate that NP-59 has too high lipophilicity for uptake into the liver via these transporters. Although human liver S9 does not contain all hepatic enzymes, NP-59 had high stability at 120 min after injection in mouse blood, mouse bile, mouse liver, and human liver (Table 1). Kojima *et al*. reported that NP-59 has higher stability than other cholesterol analogs^16^. NP-59 underwent minimal deiodination because radioactivity in the thyroid and stomach, in which accumulation indicates deiodination, was low, as seen in the biological distribution study of normal mice (Table 2). Therefore, we concluded that NP-59 is stable in the body and can be used to measure hepatic ABC transporter function.

NP-59 was excreted via MRP1 and BCRP (Fig. 2). MRP1 is expressed on the basolateral side of hepatic epithelial cells for export from liver to blood, whereas BCRP is expressed on the apical side of hepatic epithelial cells for excretion into bile. We observed a significant difference between BCRP and MRP1 (BCRP was lower than MRP1) for NP-59 transport (Fig. 2). Thus, efflux of NP-59 may be higher via BCRP than MRP1.

In our study investigating the biological distribution of NP-59 in normal mice (Table 2), higher radioactivity was mainly found in the blood, lung, liver, and gall bladder. Uptake of NP-59 into the liver may depend on OATP1B1 and OATP1B3, and bile secretion of NP-59 into the gall bladder may be due to BCRP. Although NP-59 accumulated in adrenal glands more than 1 day after injection^16^, the uptake of NP-59 in the liver and the gall bladder peaked by 10 min and 30 min, respectively, and then decreased (Table 2) because NP-59 was taken up into the liver, transferred to the gall bladder, and then transferred to the small intestine and bladder. Therefore, uptake into adrenal glands has little influence on measurement of hepatic functional imaging with NP-59.

Because Allen reported that mouse *Bcrp* cDNA encodes a 657-amino acid protein with 81% identity and 86% similarity to human BCRP^25^, we used mice loaded with Ko143, which is a BCRP inhibitor to confirm the bile secretion of NP-59 into the gall bladder via BCRP. Ko143 loading led to higher radioactivity of NP-59 in the blood, brain, liver, and kidney (Table 3). BCRP is expressed not only in the liver but also in the kidney on the apical side^26^. Liver-to-blood, gall bladder-to-blood, and kidney-to-blood ratios were calculated to decrease the influence of radioactivity in blood (Table 4). Ko143 loading increased NP-59 accumulation in the liver, and decreased the accumulation in the gall bladder due to inhibition of BCRP. No influence was seen on the accumulation in kidney.

**Table 4:** Organ-to-blood ratios at 5, 10, 30, and 60 min after NP-59 injection with and without Ko143 loading.

Time (min)	5	10	30	60
**Liver-to-blood**				
NP-59 without Ko143	0.10 ± 0.02	0.10 ± 0.02	0.24 ± 0.03	0.25 ± 0.05
NP-59 with Ko143	0.10 ± 0.01	0.14 ± 0.02*	0.27 ± 0.03*	0.23 ± 0.05
**Gall bladder-to-blood**				
NP-59 without Ko143	0.09 ± 0.01	0.13 ± 0.02	0.41 ± 0.05	0.27 ± 0.05
NP-59 with Ko143	0.06 ± 0.01	0.09 ± 0.02*	0.27 ± 0.05**	0.24 ± 0.04*
**Kidney-to-blood**				
NP-59 without Ko143	0.02 ± 0.00	0.02 ± 0.00	0.03 ± 0.00	0.04 ± 0.00
NP-59 with Ko143	0.02 ± 0.00	0.02 ± 0.00	0.04 ± 0.00	0.04 ± 0.00

All data are the mean ± standard deviation measured in five mice.

**P < 0.01 and *P < 0.05 between NP-59 without Ko143 and with Ko143 loading.

Although planar imaging with NP-59 can usually be applied for detection of adrenal adenomas and hyperaldosteronism in clinical adrenal gland scintigraphy, we estimate that SPECT/CT imaging can be useful to separate the gall bladder and right adrenal gland because the radioactivity of NP-59 in the gall bladder may be confused for that in the right adrenal gland. In SPECT imaging (Fig. 3), NP-59 accumulated in the lung, liver, and gall bladder in normal mice. In mice with Ko143 loading, accumulation of NP-59 in the liver was increased, whereas accumulation in the gall bladder was slightly decreased compared with normal mice. The ratios of radioactivity in the gall bladder to the liver were significantly different between normal mice (2.45 ± 0.45) and mice with Ko143 loading (1.60 ± 0.74). If the function of BCRP is low in clinical studies due to certain diseases, the ratios will be significantly decreased compared to healthy subjects with normal function of hepatic BCRP, as suggested by the results of Ko143 loading.

NP-59 is useful for measuring human hepatic function of OATP1B1, OATP1B3, and BCRP. If ^131^I labeling of NP-59 is changed to ^123^I, the imaging quality will be improved. However, NP-59 may not be the best agent for hepatobiliary scintigraphy because uptake of NP-59 into the liver and gall bladder is slower than that of other imaging agents used for hepatobiliary scintigraphy due to the possibility of a somewhat low affinity of the agent for OATP and BCRP as shown by the results of other studies^10–12^. Therefore, chemical analogs of NP-59 may have higher affinity for these transporters and thus be more suitable for use in hepatobiliary scintigraphy.

## Conclusion

NP-59, which is used clinically for adrenal gland scintigraphy, can be used to visualize and measure the function of human hepatic OATP1B1, OATP1B3, and BCRP.
