# Author Correction: Imaging of hepatic drug transporters with [^131^I]6-β-iodomethyl-19-norcholesterol

**DOI:** 10.1038/s41598-019-54992-4

**Published:** 2019-12-06

**Authors:** Masato Kobayashi, Kodai Nishi, Asuka Mizutani, Tsuzumi Hokama, Miki Matsue, Tetsuya Tsujikawa, Takeo Nakanishi, Ryuichi Nishii, Ikumi Tamai, Keiichi Kawai

**Affiliations:** 1 School of Health Sciences, Institute of Medical, Pharmaceutical and Health Sciences, Kanazawa University, Ishikawa, Japan; 2 Department of Radioisotope Medicine, Atomic Bomb Disease Institute, Nagasaki University, Nagasaki, Japan; 3 Biomedical Imaging Research Center, University of Fukui, Fukui, Japan; 4 School of Pharmaceutical Sciences, Institute of Medical, Pharmaceutical and Health Sciences, Kanazawa University, Ishikawa, Japan; 5 Faculty of Pharmacy, Takasaki University of Health and Welfare, Gunma, Japan; 6 Department of Molecular Imaging and Theranostics, National Institute of Radiological Sciences, Chiba, Japan

Correction to: *Scientific Reports* 10.1038/s41598-019-50049-8, published online 16 September 2019

The original version of this Article omitted Ryuichi Nishii and Masato Kobayashi as equally contributing authors. This has now been corrected in the PDF and HTML version of the paper.

